# Management and Prognosis of Physical Therapy for the Post-Surgical Sequelae of Metastatic Cervical Lymphadenopathy

**DOI:** 10.7759/cureus.27673

**Published:** 2022-08-04

**Authors:** Sakina S Saifee, Shubhangi P Patil, Rupali B Thorat, Shivani S Lalwani, Tasneem M Lakkadsha

**Affiliations:** 1 Department of Physiotherapy, Ravi Nair Physiotherapy College, Datta Meghe Institute of Medical Sciences, Wardha, IND

**Keywords:** quality of life, squamous cell carcinoma, mouth opening, physiotherapy, metastatic cervical lymphadenopathy

## Abstract

A common secondary complication of oral malignant carcinoma is metastatic cervical lymphadenopathy. The condition is typically treated surgically, with the affected cervical lymph nodes excised, followed by pharmacological treatment. However, additional complications such as asymmetry of facial features, reduced mouth opening, adhesions in sutured tissues, and so on accompany surgical management. This case report describes a case of an adult male who underwent surgery for metastatic cervical lymphadenopathy caused by previous squamous cell carcinoma of buccal mucosa. To address the surgical outcomes that were affecting the patient's quality of life, an integrated physiotherapy management protocol was developed and efficiently followed for three weeks. Improvements in mouth opening, tongue movement, cervical joint movement, chest movement, and the Oral Health Impact Profile quality of life questionnaire were observed at the three-week evaluation, indicating that the intended therapy was effective.

## Introduction

Lymph nodes that are irregular in size, consistency, or number are referred to as lymphadenopathy [1]. The lymph nodes in the neck account for approximately 40% of all the lymph nodes in the human body. Cervical lymph node lesions vary in nature and have a malignancy rate of more than 50%. Metastatic carcinoma and lymphoma are the most common cervical malignant lesions, with cervical lymph node metastatic carcinoma accounting for around 3/4 of all malignant neck lesions [2]. The majority of cervical malignant lesions (85%) are primary head and neck lesions, with thyroid carcinoma (TC), salivary gland carcinoma (SC), and squamous cell carcinoma (SCC) being the most common nodal metastases [3]. Buccal mucosa squamous cell carcinoma is an uncommon and aggressive form of oral cavity cancer [4]. It's also linked to a high likelihood of local-regional recurrence, and it is most common in people who chew tobacco and/or smoke regularly, whether or not they drink alcohol [5].

A middle-aged male was diagnosed with metastatic cervical lymphadenopathy after a history of two recurring squamous cell carcinomas of the buccal mucosa. He was treated surgically and pharmacologically for carcinoma twice, but the rehabilitation component of the post-reconstruction management was ignored each time. Although surgery helps to remove the malignant tumor, it also leaves the patient with structural asymmetry, sutures and scars, edema, a lack of mental and social self-confidence, and other negative effects, which we refer to as surgical aftereffects. The patient’s most recent operation for lymphadenopathy contributed to the prior ones' aftereffects, restricting his mouth opening, tongue movement, and cervical joint mobility. A comprehensive physiotherapy plan was required to counter the effects of the surgeries and enhance his quality of life as much as possible.

## Case presentation

A 43-year-old male with a known case of squamous cell carcinoma of the right buccal mucosa underwent surgery followed by cardiotoxicity of radiation therapy (CTRT), and now on follow-up, complained of swelling and pain on the left side of the neck area for the last 20 days. On examination, a single left middle jugulodigastric lymph node was palpable, size 4x3 and being roughly oval, hard, fixed, and painful. He received ultrasound sonography-guided fine needle aspiration (USGFNA) for the cytologic investigation of suspicious lymph nodes and 18F-fluorodeoxyglucose (18F-FDG) PET-CT in which PET using 18F-FDG, an analogue of glucose, reveals metabolic abnormalities before morphological changes emerge and precise anatomical location of lesions [6]. Both the tests confirmed the diagnosis of metastatic left cervical lymphadenopathy, which was followed by left side modified radical neck dissection surgery.

The patient had already undergone two consecutive oral surgeries for well-differentiated squamous cell carcinoma previously. He had wide local excision of the lesion with modified radical neck dissection (MRND) type II and reconstruction with buccal fat in the right oral mucosa at a private hospital 19 months ago. He had a second procedure nine months later at the same clinic, which comprised a large local excision with extended marginal mandibulectomy and repair using a right pectoralis major myocutaneous (PMMC) flap. The patient got 30 fractions of radiation treatment and six rounds of chemotherapy within two months following the second operation. The patient had a habit of chewing tobacco 3-4 times a day for the past 25 years, which he continued after his first carcinoma surgery.

The patient was examined post-operatively and found to be mesomorphic with a BMI of 26.78 kg/m^2^. His vital signs were normal, with an 80-beat-per-minute pulse, an 18-breath-per-minute respiratory rate, and a blood pressure of 120/80 mmHg. During the examination, there was significant asymmetry throughout the face, with an adherent scar on the right side from previous surgery. Edema was present in the right neck region. A 10-cm long suture extended from the thyroid cartilage to the sternocleidomastoid muscle across the neck. The suture site was kept covered with a bandage.

**Figure 1:**
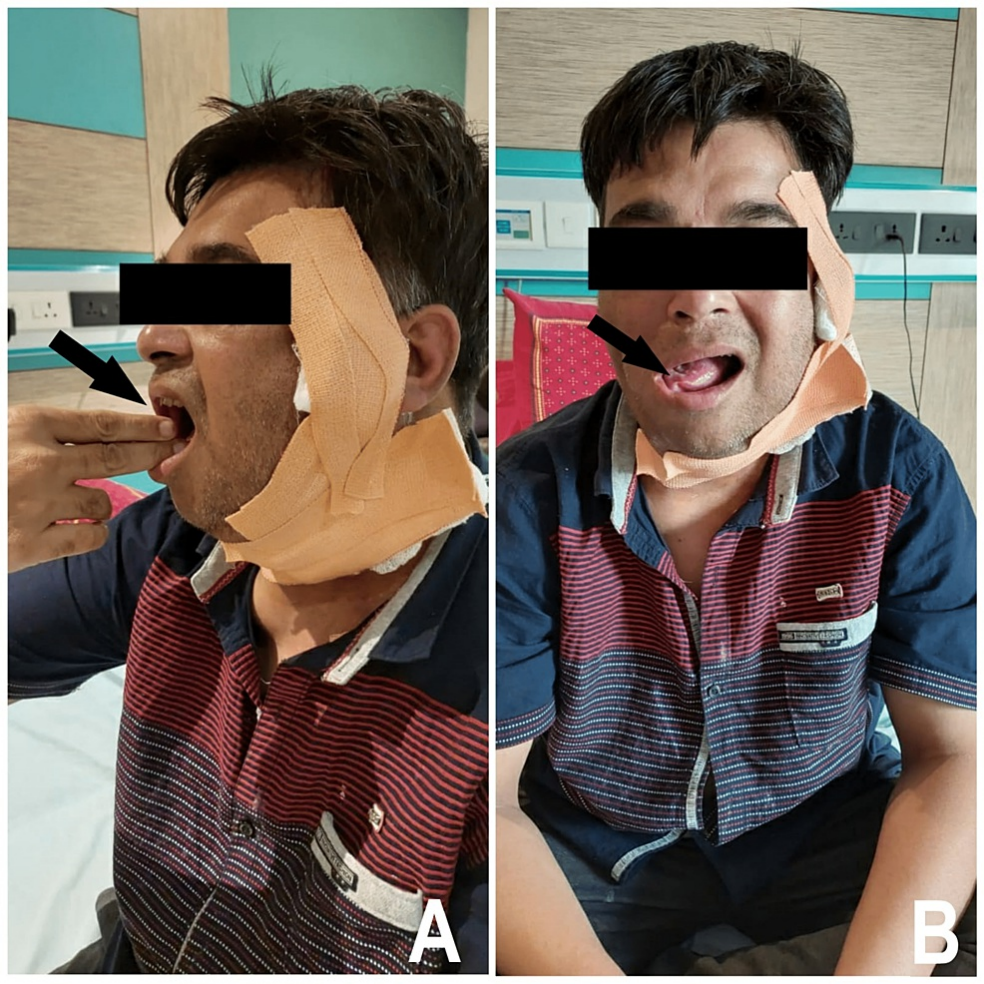
The Patient is Seen Demonstrating (A) Restricted Mouth Opening and (B) Reduced Tongue Movement Towards Left Side

Palpation corroborated the observatory findings. The cervical edema was pitting in appearance, with grade II indentation. The patient's mouth opening was reduced to 30 mm (2 and 1/2 finger width approx.) during intra-oral examination, as indicated in Figure 1A and Figure 1B. On both sides, the jaw movements were smooth and synchronous. Tongue movement to the left was reduced (Figure 1B). The patient faced drooling of saliva and a little difficulty in speech due to the pain at suture site and reduced tongue movement. Due to the prior surgery's flap taken from the pectoralis major muscle, which induced stiffness in the right chest, and the latest surgery's excision of the left sternocleidomastoid muscle, chest expansion was limited. Bilaterally, cervical joint movements such as lateral flexion and rotation were similarly reduced.

Soon after the procedure, physiotherapy treatment began. The objectives were based not just on the recovery from the most recent surgery, but also on the impairments from the previous surgery. The treatment plan was divided into two parts: acute for the first week and progressive for the next two weeks. Table 1 highlights the objectives and interventions used in both phases to achieve them. Each intervention's regimen is listed in the same table. Based on the improvement seen on the end measures, interventions were included or substituted in the second phase.

**Table 1:** Physiotherapy Management Plan TMJ: Temporomandibular joint; CRAC: Contract-Relax, Antagonist-Contract; PNF: Proprioceptive Neuromuscular Facilitation; AROM: Active Range of Motion

Sr. No.	Goals	Therapeutic Interventions	Regimen
Summary of Acute Phase Protocol (week 1)			
1.	To psychologically prepare the patient for the rehabilitation	The patient education regarding his present health condition and the importance of the prescribed protocol. Motivation for rehabilitation adherence.	At the beginning of the protocol and whenever the patient felt unmotivated.
2.	To initiate the mouth opening	Stick exercises for mouth opening	Exercise thrice a day. Increase the number of sticks as per the progression
		Active range of motion exercises for the TMJ in all planes	A set of 10 reps thrice a day.
		Chin tucks	
3.	To improve the tongue movements	Tongue exercises included tongue-hold maneuver, isometric exercises using a tongue depressor.	A set of 10 reps thrice a day.
		Tongue movements in all the directions	Tongue ROM exercises are performed as many times as possible throughout the day.
4.	To loosen the adhered tissue of the cheek scar	Local squeezing, stretching, finger & thumb kneading at the adhered tissue of the right cheek.	Therapy for 5-7 minutes once a day
5.	To improve cervical joint movements	Active ROM exercise for the cervical joint in every axis	A set of 10 reps thrice a day
		Isometric strengthening exercises	
6.	To reduce the swelling in the cervical region	Effleurage therapy	Effleurage therapy for 3-5 minutes once a day.
		AROM exercises	A set of 10 reps thrice a day
7.	To improve the pectoralis major muscle activation of the right side for symmetrical thoracic movement	Breathing exercise- Thoracic expansion exercises	Two sets of 5 reps- twice a day
8.	To increase the shoulder joint mobility	Full range shoulder mobility exercise	A set of 20 reps thrice a day
		Scapular movements	
Summary of Progressive Phase Protocol (week 2 and 3)			
1.	To achieve the maximum possible mouth opening	Stick exercises for mouth opening	Exercise thrice a day. Increase the number of sticks as per the progression
		Active jaw stretching exercise	A set of 10 reps twice a day.
		CRAC (“Contract-Relax, Antagonist-Contract”) technique	
2.	To regain functional tongue movements	Tongue resistance, tongue retraction, strengthening exercises	A set of 10 reps thrice a day
3.	To improve the facial symmetry	Stretching of the elevator and depressor muscles	A set of 10 reps twice a day
		PNF exercises for facial muscles	
		Therapeutic ultrasound	Frequency-3 MHz, given for 7 minutes once daily.
4.	To normalize the range of movement and strength of the cervical joint	Strengthening with increasing repetitions and ranges	A set of 10 reps twice a day.
5.	To increase the flexibility of the pectoralis major	Pectoral stretching exercise	A set of 20 reps twice a day
6.	To regain normal mobility at the shoulder joint	Shoulder joint stretches	A set of 10 reps twice a day
		Shoulder joint strengthening using weight cuffs	2 sets of 20 reps with 1 kg weight- twice a day.
7.	Home exercise program	Counseling the patient to build self-confidence to face the society	At the time of discharge
		Advice to follow the above protocol at home.	
		Start with chewing light to hard substances from the affected side.	
		Follow update suggested	

The physiotherapy treatment lasted three weeks throughout the post-operative in-patient period, after which the patient was discharged with a well-explained home exercise programme. The patient was thoroughly examined using outcome measures prior to discharge. Table 2 shows the positive findings for the outcomes.

**Table 2:** Treatment Outcomes Measured Before and After the Treatment ROM: Range of motion

Sr. No.	Outcomes	Before treatment			After treatment (post 3 weeks)		
1.	Mouth opening	30 mm (2 and ½ finger)			40 mm (3 complete fingers)		
2.	Tongue deviation (Grading scale- functional classification of ankyloglossia)	Grade IV - No deviation to the left side from the midline			Grade III - <50% of the normal tongue range		
3.	Cervical joint ROM	Lateral flexion	Left	30^o^	Lateral flexion	Left	40^o^
			Right	20^o^		Right	35^o^
		Rotation	Left	40^o^	Rotation	Left	65^o^
			Right	40^o^		Right	70^o^
4.	Chest expansion level						
	Axillary	1 cm/3 cm			2 cm/3 cm		
	Nipple	2 cm/5 cm			3 cm/5 cm		
	Xiphisternum	5 cm/7 cm			5 cm/7 cm		
5.	Oral health impact profile - 14 (The larger the score, more the disability)	36/56			25/56		

## Discussion

Lymphadenectomy is the surgical removal of one or more groups of lymph nodes [7]. A 43-year-old man with recurrent malignant squamous cell carcinoma of the buccal cavity was recently operated on for malignant cervical lymphadenopathy and was referred to a physiotherapist for complaints of restricted mouth opening with reduced movements at the tongue, cervical joint, temporomandibular joint, and chest movement. Only a few approaches had been shown to be beneficial in this condition, but a comprehensive treatment plan was necessary for a full recovery. As a result, we endeavored to merge these different approaches with the others in order to create an integrated therapeutic plan.

Tissues and scars that had become adhered to the underlying structures post-surgery needed to be released in order to promote mouth opening and improve facial symmetry [8]. The use of a therapeutic ultrasonic device in combination with finger and thumb kneading in the buccal region loosens adherent fibrous tissue due to collagen fiber dissociation and softening of cement components, resulting in enhanced tissue elasticity [9,10]. One of the biggest challenges the patient had was limited tongue movement, which made speaking and swallowing difficult. Tongue exercises such as tongue resistance, retraction, tongue-hold maneuver, tongue ROM, and strengthening exercises were recommended. These exercises helped in increasing the mobility as well as the flexibility of the tongue musculature, which further aided in molding the tongue as per the speech. The mouth opening exercises with sticks started the day following the surgery as they improve oral opening by remodeling the tissues. As therapeutic measures, labial, lingual, jaw, neck, mandibular, and shoulder range of motion (ROM) exercises, as well as jaw resistance techniques, were included to maintain their structural and functional properties [11]. An earlier research recommended a four-month physiotherapy program for these types of conditions [12], but this case report provides a detailed week-by-week care plan for the same group. The three-week in-patient therapy was followed by a telephone-supervised home management programme. The efficacy of the procedure was proven by the findings of the outcome measures.

## Conclusions

This case report provides an integrated detailed week-by-week physiotherapy rehabilitation programme for patients who developed cervical lymphadenopathy following metastatic oral surgery. When the before and after treatment outcomes were compared, the therapy plan provided favorable results, which will help such individuals recover sooner in the future. The commencement of the protocol early after the surgery aids in the patient's recovery from the surgery's secondary effects and allows him to soon regain his social life.
